# Cross-system effects of dysphagia treatment on dysphonia: a case report

**DOI:** 10.1186/1757-1626-1-67

**Published:** 2008-07-30

**Authors:** Lisa A LaGorio, Giselle D Carnaby-Mann, Michael A Crary

**Affiliations:** 1 Department of Communicative Disorders, College of Public Health and Health Professions, PO Box 100174, University of Florida, Gainesville, FL, 32610-0174, USA; 2 Department of Behavioral Science and Community Health, College of Public Health and Health Professions, PO Box 100175, University of Florida, Gainesville, FL, 32610-0175, USA

## Abstract

Traditionally, treatment of dysphagia and dysphonia has followed a specificity approach whereby treatment plans have focused on each dysfunction individually. Recently however, a therapeutic cross-system effect has been proposed between these two dysfunctions. At least one study has demonstrated swallowing improvement in subjects who completed a dysphonia treatment program. However, we are unaware of any evidence demonstrating the converse effect. In this paper, we present a case-report of a 74 year old male who demonstrated improvement in selected vocal parameters after completion of a dysphagia therapy program.

Dysphagia therapy resulted in improved laryngeal function in this subject. Results implicate improved vocal fold tension with increased glottal closure. Further investigation into the potential for this cross-system effect is warranted.

## Introduction

Swallowing and phonation represent different functions of the aerodigestive tract that share a common subsystem. Consequently, an injury or disease process that impairs either of these functions has the potential to impair the other. Traditionally, therapy programs adhered to a treatment specificity principle addressing these dysfunctions as separate concerns. However, recent evidence suggests that a cross-system interaction may exist between them [1], implying that the treatment specificity principle may not be essential in rehabilitating either dysphagia or dysphonia. One study has demonstrated swallowing improvement in subjects who completed a dysphonia treatment program [2]; however, we are unaware of any evidence demonstrating the converse effect.

Exploring potential cross-system treatment effects between dysphagia and dysphonia is appropriate among patients with head and neck cancer, as both disorders may occur concomitantly in 44% to 47% of this patient population [3,4]. This case-report documents voice improvement during dysphagia therapy in one head and neck cancer patient.

## Case Presentation

A 74-year old, retired, white male with treatment refractory dysphagia following chemo-radiation therapy for treatment of a T3N1M0 base of tongue squamous cell carcinoma, enrolled in an experimental dysphagia treatment research program at a university-affiliated speech and hearing clinic. Nine months prior to initiating this dysphagia therapy program he completed 40 doses of radiation therapy (7200 cGray to the primary tumor site and 5040 cGray to a secondary neck lymphnode) in conjunction with cisplatin chemotherapy. Following chemoradiation he completed two separate courses of dysphagia therapy. At time of enrollment in this dysphagia treatment program, he received nearly all nutrition and hydration via percutaneous gastrostomy tube. Daily oral intake was limited to approximately two ounces of pureed foods. Vocally, he complained of fatigue, pitch breaks, difficulty being understood on the telephone, and reduced singing ability.

He completed 15 days of an experimental swallowing exercise program supported with transcutaneous neuromuscular electrical stimulation (NMES) [5]. Swallowing exercise involved performing the effortful swallow technique while ingesting a progressive hierarchy of liquids and solids. NMES was delivered via the VitalStim^® ^NMES unit (Chattanooga Group, Hixson, TN). The VitalStim device uses two pairs of electrodes placed vertically along the midline of the anterior neck to supply the electro-stimulation.

On treatment day five, perceptual changes in the subject's voice were noted, prompting daily acoustic measurements of three vocal parameters beginning on treatment day six. Acoustic measurements of maximum phonation time (MPT), pitch range (highest and lowest attainable pitch), and habitual pitch while reading were obtained using the VisiPitch IV (KayPentax: Lincoln Park, NJ). Each parameter was measured three times before and after all therapy sessions and during the three follow-up sessions. Mean and standard deviation of the three trials were calculated for each task. A total of 10 treatment and three follow-up sessions were recorded.

Pre-, post, and follow-up swallowing function was evaluated via standardized clinical, endoscopic, and videofluoroscopic evaluations including completion of the Mann Assessment of Swallowing Ability (MASA) [6] and the Functional Oral Intake Scale (FOIS) [7], as well as self perception of swallow function measured via bisection of a 100 mm visual analog scale (VAS). Each voice parameter was analyzed using repeated measures ANOVA with Bonferroni correction for multiple comparisons. When significant interaction or main effects were identified for any voice parameter, Tukey pairwise post-hoc analysis was employed. Of primary focus were comparisons made between baseline vocal measurements (session 6) and end of treatment measurements (session 15).

**Figure 1:**
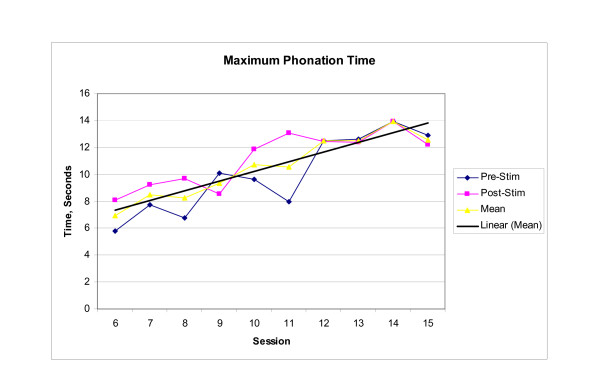
**Maximum Phonation Time**. The longest length of continuous phonation produced while sustaining/a/at a comfortable pitch and volume. Each Pre-stim and Post-stim data point represents the mean of three trials of the task. The mean data point represents the mean performance during each session. The trend line represents the change in session mean score across the treatment sessions.

**Figure 2:**
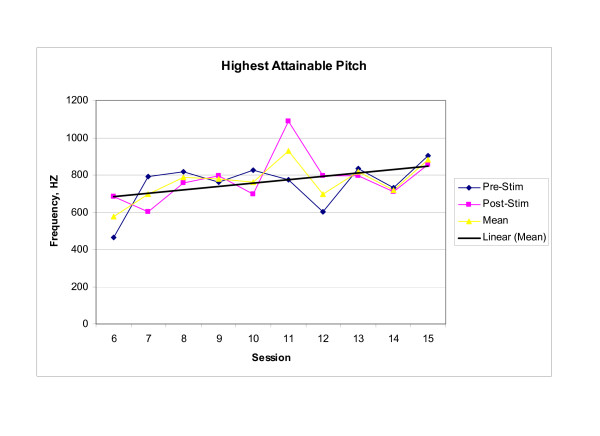
**Highest Attainable Pitch**. The highest attainable pitch produced without straining, while sustaining/a/in an upward glissando. Each Pre-stim and Post-stim data point represents the mean of three trials of the task. The mean data point represents the mean performance during each session. The trend line represents the change in session mean score across the treatment sessions.

**Figure 3:**
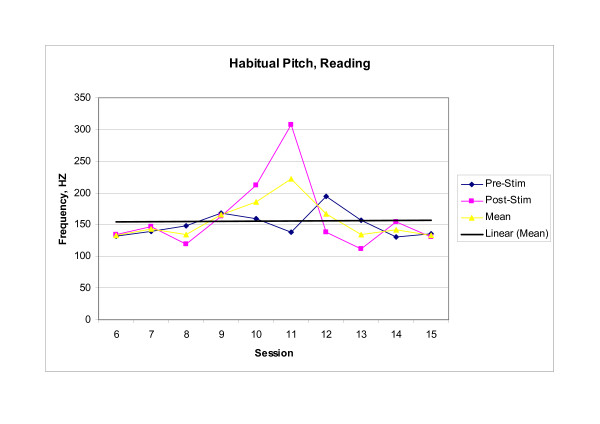
**Habitual Pitch**. Subject's habitual pitch produced while reading the first sentence of the Rainbow Passage. Each Pre-stim and Post-stim data point represents the mean of three trials of the task. The mean data point represents the mean performance during each session. The trend line represents the change in session mean score across the treatment sessions.

Endoscopic, perceptual, and instrumental vocal results supported the existence of a cross-system interaction between dysphagia rehabilitation and improved laryngeal function. Baseline endoscopic examination revealed significant supraglottic compression and a glottal gap during phonation, while post therapy examination revealed decreased supraglottic compression and glottal closure during phonation. Perceptually, the subject reported that his voice was louder, that he was able to sing in church again, and that others reported being able to better understand him on the telephone. Instrumentally, significant between session main effects were observed in both MPT (F_(9,20) _= 7.993, p < .001) and highest attainable pitch (F_(9,20) _= 3.620, p = .008). Significant within session interaction effects were observed in habitual pitch [(F_(9,20) _= 14.215, p < .001)]. No significant effects were observed for lowest attainable pitch (F_(9,20) _= 0.949, p = .513). Mean scores for those voice parameters demonstrating significant change within or across treatment sessions are presented graphically in Figures 1, 2, 3. Scores for baseline, post therapy, and follow up measures for each vocal parameter are shown in Table 1.

**Table 1:** Average Performance for Each Vocal Parameter at Baseline, Post Treatment and During the Three Follow-up Sessions.

**Vocal ** **Parameter**	**Session 6** **Pre-Tx ** **Mean** ** (SD)**	**Session 15** **Post-Tx ** **Mean** ** (SD)**	**1-Wk f/u** **Mean** ** (SD)**	**1-Mo f/u ** **Mean** ** (SD)**	**6 Mo-f/u** **Mean** ** (SD)**
MPT (sec)	5.79 (0.19)	12.2 (0.37)	13.94 (1.94)	10.70 (0.13)	13.58 (0.88)
Highest Attainable Pitch (Hz)	464.40 (167.84)	857.99 (263.5)	832.56 (114.30)	860.32 (287.20)	378.98 (23.27)
Lowest Attainable Pitch (Hz)	50.32 (0.36)	51.90 (1.22)	50.27 (0.24)	52.4 (2.34)	71.09 (0.81)
Habitual Pitch (Hz), Reading	131.77 (9.08)	130.74 (13.01)	121.84 (4.52)	139.13 (6.50)	106.22 (6.83)

Since comparisons between the baseline measurements (session 6) and the end of treatment measurements (session 15) were of primary focus, post hoc analysis was completed for MPT, highest attainable pitch, and habitual pitch. Analysis revealed that mean MPT increased significantly between session 6 and session 11 (p = .003) with this increase maintained through session 15 and the three follow-up sessions; mean highest attainable pitch increased significantly between treatment sessions 6 and 11 (p = .004), and demonstrated a trend toward significance between sessions 6 and 15 (p = .017) with the increase maintained through the one-week and one-month follow-up sessions, but not at six months post treatment. Furthermore, the increase in habitual pitch noted in session 11 was significant when compared to both baseline session 6 (p < .001) and last treatment session 15 (p < .001), but no significant change was noted between sessions 6 and 15 (p = 1.00).

This patient demonstrated improvement in all swallowing measures immediately after completion of the therapy program (Table 2). Total oral intake increased from a few bites of pureed or soft food each day, to three to five daily meals of solid food including waffles, sandwiches, and steak, while tube feeding simultaneously decreased from 8–9 cans daily, to 2–3 cans daily. MASA score increased 19 points; an increase of 10 points has been shown to be clinically significant [6]. FOIS increased one level reflecting the increase in oral diet and simultaneous decrease in tube feeding. Lastly, the subject self-reported a 77 mm increase in self-perception of swallowing ability on the VAS. Swallowing improvements observed immediately post therapy were not maintained through the 6-month follow-up due to post-radiation changes, specifically radionecrosis of his mandible and complications affecting his esophagus. Consequently, by the sixth month post therapy, nutrition was delivered via full enteral feeding; oral intake was limited to occasional sips of liquid.

**Table 2:** Swallowing Outcome Measures

**Swallowing Measure**	**Pre Therapy**	**Post ** **Therapy**	**6 Month** ** Follow-up**
FOIS	2	3	2
MASA	176	195	189
VAS	15 mm	92 mm	18 mm

## Conclusion

This case-report documented improvement in MPT and highest attainable pitch in one individual who completed a dysphagia therapy program. Although phonation and swallowing represent two different laryngeal functions, the improvements seen in this case report support recent evidence that treating deficits in one function may result in cross-system effects on the function not being actively rehabilitated [1].

One interpretation from this case is that this dysphagia therapy program improved laryngeal muscle functioning. Since the dysphagia therapy included swallowing exercise paired with concurrent transcutaneous electrical stimulation over the larynx, either, or both of these treatment components may have resulted in improved laryngeal function. Swallowing exercise incorporated the "effortful swallow" technique which has been shown to prolong elevation of the larynx pre-swallow [8], and increase duration of laryngeal vestibule closure during the swallow [9]. The cumulative effect of repetitively producing an effortful swallow in combination with advances in the oral diet may have improved laryngeal muscle function during the therapy period. Conversely, reducing oral intake during the follow-up period may have led to a muscular detraining effect and subsequent regression in laryngeal muscle functioning necessary for maintaining higher pitch levels. Furthermore, application of transcutaneous electrical stimulation directly over the larynx likely facilitated contraction of the superficial cricothyroid muscles. Enhanced contraction of these muscles would result in increased vocal fold tension and improved glottal closure.

Timing of the observed vocal changes supports the impact of the treatment program on laryngeal functioning. Voice changes were initially noticed at the end of the first week of dysphagia therapy. Next, significant increase in all voice parameters was noted at session 11, a time point corresponding with an increase in therapy program difficulty; e.g., increase in laryngeal exercise. Last, deterioration in vocal function noted at six months corresponded clinically with a decline in swallowing function, possibly reflecting a decrease in laryngeal maintenance exercise associated with a reduction of swallowing activity. When the timing of improvement and decline in voice characteristics is considered in conjunction with the improvement and decline in swallowing function, one can speculate that the combined effect of intensive exercise and NMES contributed to an underlying increase in laryngeal muscle function.

This case-report raises important clinical questions regarding treatment effects which must be tested in future controlled studies. Future studies of potential cross-system effects should include replication in larger samples, across different diagnoses, with appropriate control conditions. Appropriate controls would include assessment of respiratory capacity before/following treatment, separation of exercise vs. NMES effects, and comparison to traditional voice therapy techniques.

To our knowledge, this report is the first to document improvements in voice function during dysphagia therapy, supporting emerging evidence for a potential cross-system rehabilitative effect between dysphagia and dysphonia. The pattern of change observed in this case suggests a positive impact of intensive dysphagia therapy combined with transcutaneous NMES on laryngeal muscle function. Observations made in this case report suggest a new and interesting program of clinical research.

## List of Abbreviations

NMES: Neuromuscular electrical stimulation. MPT: Maximum phonation time. MASA: Mann Assessment of Swallowing Ability [6]. FOIS: Functional Oral Intake Scale [7]. VAS: Visual analog scale. ANOVA: Analysis of variance.

## Competing interests

The VitalStim^® ^device and all electrodes used to deliver the electrical stimulation were supplied by the Chattanooga Group (Hixson, TN). At the time of this study, Dr. Crary was the recipient of an educational grant from Chattanooga Group to support general research efforts in this area.

## Authors' contributions

LL treated the subject, analyzed the data under GC-M's supervision, and wrote the draft of the manuscript. GC-M and MC developed the treatment protocol, conceptualized the study, supervised the data analysis, and edited the manuscript. Additionally, MC completed baseline and follow-up dysphagia evaluations and supervised LL (a PhD student) in all aspects of the case study. All authors read and approved the final manuscript.

## Consent

All procedures used in this experimental dysphagia treatment protocol were approved by the local Institutional Review Board. The subject signed an informed consent prior to initiating the dysphagia therapy protocol.
